# Suicidal vulnerability in older adults and the elderly: study based on risk variables

**DOI:** 10.1192/bjo.2022.42

**Published:** 2022-04-01

**Authors:** David Sánchez-Teruel, María Auxiliadora Robles-Bello, Aziz Sarhani-Robles

**Affiliations:** Department of Personality, Assessment and Psychological Treatment, University of Granada, Spain; Department of Personality, Assessment and Psychological Treatment, Spanish Society of Suicidology, Spain; and Department of Personality, Assessment and Psychological Treatment, Spanish Society of Clinical and Health Psychology, Spain; Department of Psychology, University of Jaen, Spain; Faculty of Medicine, University of Granada, Spain

**Keywords:** Suicide attempt, risk, epidemiology, healthcare protocols, old age

## Abstract

**Background:**

Predicting suicidal vulnerability based on previous risk factors remains a challenge for mental health professionals, especially in specific subpopulations.

**Aims:**

This study aimed to use structural equation modelling to assess which sociodemographic and clinical variables are most predictive and modulating of repeated self-injury or reattempts at suicide in older adults and the elderly with previous attempts.

**Method:**

We obtained digital data for 619 people (*N* = 342; 55.3% women), aged 50–96 years (mean 71.2 years, s.d. 3.65), who presented to the emergency department with a repeated self-injury or suicide attempt. Data were collected from several public and private hospitals in southern Spain.

**Results:**

There were different sociodemographic and clinical profiles between people who repeat self-injury and those who reattempt suicide. In addition, we show that outcome variables may directly or indirectly modulate these behaviours.

**Conclusions:**

The study findings provide only a limited insight into suicidal vulnerability in older people, and there is an urgent need for specific care protocols for the prevention of repeated self-injury or reattempts at suicide that are adapted to the psychosocial characteristics of this age group. There is also a need to improve social and health alert actions for older adults and the elderly who present with suicide risk profiles, and the presence of mental health professionals in hospital emergency departments should be improved.

## Suicide on eldery people

Suicide rates have focused primarily on fatal outcomes,^1^ and there are few studies on attempts. However, data indicate that there are 25 attempts for every death from suicide,^2^ and to date, attempting suicide has been identified as the only risk behaviour that predicts future fatal reattempts and death from suicide.^3^ In addition, there are specific populations such as older adults and the elderly who show distal signs of significant risk, such as self-injury, as a prelude to attempting suicide.^4^ Suicide attempts are often associated with the intention to die, unlike self-harm, which is characterised by the absence of lethal intent.^3–5^ Both behaviours are very frequent in this subpopulation, where individuals consciously self-injure,^1^ which could increase the risk of repetition and early death.^5^ However, data on the risk variables that modulate or predict the two outcomes are very scarce.

Female suicide does not tend to vary much throughout life, whereas male suicide rates are markedly higher in older people than in other age groups.^6^ In addition, there are other sociodemographic factors that appear to modulate the risk (increasing or decreasing it) for suicide attempts in older adults and the elderly. Examples include marital status and the method used, or clinical factors such as pre-existing physical pathologies or a mental disorder.^7^

## Risk and protective factors

Suicide methods are also key to drawing up a risk profile.^8^ Self-poisoning and firearms are the most commonly used methods in completed suicides in older adults.^9^ In addition, this stage of life also brings changes in physical and mental health and a process of socioeconomic adaptation resulting from retirement.^10^ This can lead to older and elderly people having reduced autonomy and independence, which can amplify self-perceptions of being a burden on family and friends.^11^ These aspects have the potential to modulate how self-injuries and suicide attempts are made.^5^ However, the variability of suicide risk factors in older adults continues to provide very heterogeneous data, making the prevention of suicide difficult.^12^ Furthermore, few studies have analysed a vulnerability profile for future reattempts at suicide in older adults and the elderly with previous self-injuries or suicide attempts, indicating a pressing need for further research.^13^

Therefore, this study aimed to assess what sociodemographic and clinical variables were more predictive of future self-injuries or reattempts at suicide in older adults and the elderly with previous self-injuries or previously attempted suicide.

## Method

### Participants

The total sample was 619 (non-institutionalised) people from the community (*N* = 342; 55.3% women), aged 50–96 years (mean 71.2 years, s.d. 3.65), selected from the pool of patients presenting to the emergency departments of several public and private hospitals in a southern province of Spain. Inclusion criteria were age ≥50 years and a diagnosis recorded by the emergency department as ‘self-inflicted injury’ or ‘suicide attempt’ between 1 January 2014 and 31 December 2018. The exclusion criteria were age <50 years, having both diagnostic categories recorded in the emergency department record (self-injury and suicide attempt) or not clearly including the medical history number in the digital report. Two groups were produced from the total sample: those aged 51–94 years (mean 57.4, s.d. 4.12) with a second episode of self-harm (*n* = 38; 31.9% women), and those aged 50–96 years (mean 71.2, s.d. 3.65) with a second suicide attempt (*n* = 81; 68.07% women). The time between the first and second self-harm attempt was between 1 and 3 months (mean 2.3 months, s.d. 1.94), and the time between the first and second suicide attempt was between 5 and 11 months (mean 7.9 months, s.d. 3.6).

### Instruments and procedures

The necessary permissions were obtained for collecting digital information from the emergency departments in several healthcare districts through their referral hospitals. Data were collected by hospital personnel through the regional digital medical records system DIRAYA (an Arabic word meaning knowledge in progress). The DIRAYA system is used in the Andalusian Health Service in Spain to maintain electronic health records and manage the health system. It includes all of the health information for everyone treated in health centres and hospitals, so that the data is available wherever and whenever they are treated. The collaborating healthcare staff recorded the sociodemographic and clinical data for patients who met the inclusion criteria outlined in the participants section. This was done over a 6-month period, depending on their availability. Approval was obtained from the Research Ethics Committee of the University of Jaen, Spain, and the Health Research Bioethics Committee of the Government of the South of Spain (approval number CEIH 031213-8). The aim was to collect digital data from emergency departments, so no consent from the participants was required.

### Data analysis

Missing data were replaced with the mean or mode imputation method.^14^ The normal distribution of the data was examined with the Kolmogorov–Smirnov test (*P* > 0.05). The multiple logistic regression with intro method^15^ was applied to measure the most predictive sociodemographic and clinical variables of repetition of self-injuries and reattempts at suicide through a two-step process:^16^ linear regression analysis was used in separate models, and then the effect of modulation of the most predictive variables was analysed by precoding the multicategorical variables in a dummy form and using path analysis. This method is used to determine the indirect and direct proportion of the total effect of independent variables on the dependent variables.^17^ The bootstrapping method was used with 10 000 resamples and an estimated 95% confidence interval to examine the significance of indirect effects. The bootstrapping method is more effective for research with a relatively small sample size.^18,19^ Additionally, the Bayesian Markov chain Monte Carlo algorithm was used.^20^ Statistical analysis was done with the Amos program in SPSS version 23 for Windows, and the minimum level of significance was set at *P* < 0.05.

## Results

Descriptive results for the total sample showed that the majority of participants were women (*n* = 342; 55.3%), aged 61–70 years (42.3%), widowed (31.2%), with a pre-existing physical illness (22.8%), and that most of them were discharged after medical attention for physical injuries (*n* = 328; 52.1%) (Table 1). Neither the main sample (Kolmogorov–Smirnov_619_ = 4.39, *P* < 0.05) or the two subsamples (Kolmogorov–Smirnov_81_ = 18.15, *P* < 0.05; Kolmogorov–Smirnov_134_ = 12.61, *P* < 0.05) exhibited univariate normality.

**Table 1:** Description of the sample sociodemographic and clinical data

	*n* (%)	Second episode of self-harm, *n* (%)	Second suicide attempt, *n* (%)	Student's *t-*test	Effect size, *d*
Civil status				2.44**	0.97
Single	131 (21.2)	15 (18.5)	31 (23.1)		
Married	168 (27.1)	11 (13.6)	47 (35.1)		
Separated/divorced	127 (20.5)	19 (23.5)	25 (18.7)		
Widowed	193 (31.2)	36 (44.4)	31 (23.1)		
Methods of self-injury or suicide attempt				4.76*	0.51
Single active method	302 (48.8)	37 (45.7)	68 (50.7)		
Mixed active method	317 (51.2)	44 (54.3)	66 (49.3)		
Pre-existing conditions				6.14**	0.09
Depression	136 (21.9)	16 (19.8)	31 (23.1)		
Anxiety	103 (16.6)	14 (17.3)	22 (16.4)		
Psychotic disorders	81 (13.1)	11 (13.6)	17 (12.7)		
Personality disorders	66 (10.7)	13 (16.0)	10 (7.5)		
Physical disorders	141 (22.8)	18 (22.2)	31 (23.1)		
No previous diagnosis	92 (14.9)	9 (11.1)	23 (17.2)		
Post-attempt healthcare action				3.61*	0.39
Discharge	328 (52.1)	46 (56.8)	68 (50.7)		
Admission	291 (47.9)	35 (43.2)	66 (49.3)		
Total	619 (100)	81 (100)	134 (100)		

*P < 0.05, **P < 0.01.

Results of the Durbin–Watson test (Durbin–Watson test = 1–3)^15^ indicated that the assumption of independence of errors was met for all independent variables used as criteria in both models (model 1: yes/no second episode of self-injury; model 2: yes/no second suicide attempt) (Durbin–Watson_model 1_ = 1.34−2.19; Durbin–Watson_model 2_ = 1.08−2.39). The assumption of non-multicollinearity was also met for all independent variables, as they gave variance inflation factor (VIF) values of <5^21^ (VIF_model 1_ = 1.37–2.69; VIF_model 2_ = 1.12–3.22).

Depending on the future behaviour (self-injury or suicide attempt), the predictor variables were different. The result of the statistical efficiency score (*χ*^2^ *P* < 0.05) indicates a significant improvement in the prediction of the probability of occurrence for the dichotomous dependent variable categories. The value of Nagelkerke's *R*^2^ is suitable for both models (Table 2). More specifically, the results of the beta exponential distribution (exp(β)) regression indicate that the most predictive risk variables for a second episode of self-injury in older adults would primarily be being younger (50–60 years) (exp(β) = 0.92), single (exp(β) = 0.76), using mixed methods to self-injure (blow to the head and self-injury to the neck) (exp(β) = 0.90) and having anxiety as a pre-existing disorder (exp(β) = 0.96). However, the most predictive risk variables for a second suicide attempt were being female (exp(β) = 0.90), being aged 71–80 years (exp(β) = 0.82), having depression (exp(β) = 0.95), having a physical illness (exp(β) = 0.93), using a single method of suicide attempt (ingestion of medication) (exp(β) = 0.74) and being discharged by the emergency department (exp(β) = 0.81).

**Table 2:** Regression equation values for the independent variables (sociodemographic and clinical) in older adults and the elderly

	*χ*^2^	*R*^2^_Nagelkerke_	β coefficient	s.e.	Wald statistic	Exp(β)	95% CI (exp(β))	
							Lower limit	Upper limit
Model 1 (*n*_1_ = 81)								
Age (50–60 years)	9.78**	0.175	0.08	0.05	5.32**	0.92	0.84	1.01
Marital status (single)	3.71*	0.309	0.27	0.14	3.59*	0.76	0.57	1.01
Pre-existing condition (anxiety)	13.51**	0.416	0.15	0.05	10.46**	0.96	0.78	0.98
Methods of self-injury (mixed)	11.96**	0.616	0.11	0.03	7.41**	0.90	0.84	0.96
Healthcare action (admission)	1.11*	0.041	0.32	0.11	1.07*	0.03	0.01	0.07
Model 2 (*n*_2_ = 134)								
Gender (female)	3.95**	0.101	1.12	0.06	6.21**	0.90	0.75	1.08
Age (71–80 years)	2.03*	0.282	0.14	0.23	5.12**	0.82	0.70	0.97
Marital status (widowed)	1.86*	0.375	0.19	0.46	3.97**	0.61	0.27	0.81
Pre-existing condition (depression)	11.83**	0.291	0.11	0.39	12.99**	0.95	0.23	0.98
Pre-existing condition (physical)	1.34*	0.336	0.91	0.67	1.17*	0.93	0.13	0.99
Suicide re-attempt method (single)	1.08*	0.598	0.33	0.18	3.23*	0.74	0.12	0.85
Healthcare action (discharge)	3.27**	0.754	1.36	0.21	4.11**	0.81	0.56	0.92

Model 1: older adults and the elderly who engage in more than one episode of self-injury. Model 2: older adults and the elderly who make more than one suicide attempt.

*P < 0.05, **P < 0.01.

**Fig. 1:**
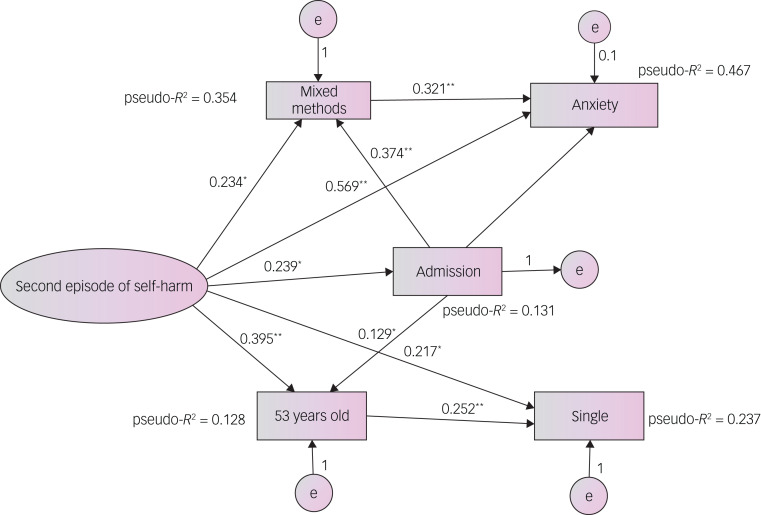
Structural equation model indicating the modulation between variables in older adults and the elderly with a second episode of self-injury. Pseudo-*R*^2^ given for categorical variables most predictive. e represents a mistake or error. *P < 0.05, **P < 0.001.

Path analyses showed that the modulating categorical variables in people with repeated self-injury episodes and reattempts at suicide were different. The results indicate that anxiety (β = 0.321, *P* < 0.01) modulates the direct effect of self-injury through an indirect variable (mixed methods for self-injury: β = 0.234, *P* < 0.1), and this indirect effect is larger than as a single variable (β = 0.569, *P* < 0.01). Indeed, the model demonstrates that anxiety indirectly mediates the relationship with self-injury through mixed methods with greater explanatory power (pseudo-*R*^2^ = 82.1%), rather than as a single direct relationship with self-injury (pseudo-*R*^2^ = 0.46.7). It also appears that emergency department admission is determined by the mixed method of injury (β = 0.569, *P* < 0.01), although this explanatory power is low (pseudo-*R*^2^ = 13.1%) (Fig. 1).

**Fig. 2:**
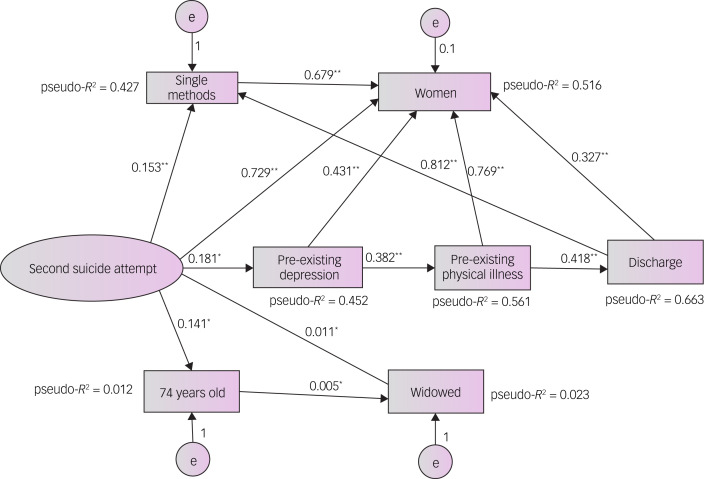
Structural equation model indicating the modulation between variables in older people with a second episode of suicide attempt. Pseudo-*R*^2^ for categorical variables most predictive. e represents a mistake or error. *P < 0.05, **P < 0.001.

On the other hand, in older adults and the elderly who make a second suicide attempt (Fig. 2), discharge from the emergency department (β = 0.418, *P* < 0.01) is very frequent if they indirectly present with pre-existing disorders such as depression (β = 0.181, *P* < 0.05) or physical illness (β = 0.382, *P* < 0.01). Additionally, the use of a single method of suicide directly determines emergency department discharge (β = 0.812, *P* < 0.01). The results also show that being female (β = 679, *P* < 0.01) with a single method of suicide attempt (β = 0.153, *P* < 0.01) indirectly modulates reattempting suicide to a greater extent than the direct relationship with gender alone (β = 0.729, *P* < 0.01). Thus, being a woman (pseudo-*R*^2^ = 51.6%) together with the presence of depression (pseudo-*R*^2^ = 45.2%) and a physical illness (pseudo-*R*^2^ = 56.1%) exhibits high explanatory power for discharge from the emergency department (pseudo-*R*^2^ = 66.3%) in this model of reattempts at suicide.

## Discussion

This study aimed to assess which sociodemographic and clinical variables were more predictive of future self-injuries or reattempts at suicide in older adults and the elderly with previous self-injuries or previous suicide attempts.

One of the main results of this study was to demonstrate differentiating clinical profiles in older people who repeat self-harm or make a second suicide attempt. The study provides important data on the role of sociodemographic and clinical variables in predicting and modulating repeated self-injury or reattempts at suicide. These results are also in line with previous research suggesting that suicide attempt rates increase with age,^22,23^ although women make more suicide attempts than men,^24^ and there is no clear gender differentiation in self-injury.^25,26^ In fact, although notable advances have been made in the study of self-injury in adolescents and young adults,^27^ there are still few results on self-injury or suicide attempts in this clinical subpopulation of older adults and the elderly with previous self-injuring behaviour.

In this study, sociodemographic factors, such as being younger (50–60 years), single, using various methods of self-harm and clinical factors such as having anxiety, were all found to be highly predictive and modulating risk variables for repeating self-harm. Repeated self-harm in older adults and the elderly has similar but distinctive characteristics to those of younger populations, which should be explored to improve the management and care of this age group, as indicated in previous studies.^5,9^ In addition, the impact of these repeated behaviours on health services is significant, as longer hospital stays are observed in this clinical subpopulation because of their previous levels of anxiety and the use of various methods of self-harm.

On the other hand, being female, older (71–80 years), widowed, having a previous physical illness and having depression were highly predictive of and modulated repeat attempts at suicide. These results showed that not only are there differences with other populations, as other studies have suggested,^28^ but that there are also differentiating sociodemographic and clinical profiles according to age, depending on the outcome (repeated self-injury or reattempts at suicide). Moreover, the risk behaviour (self-injury or suicide attempt) has been shown to produce different healthcare action in the emergency department. The results of this study show that repeated self-injury tended to result in admission, but reattempt at suicide tended to result in discharge. Once in-patient care ends, the main task for patients with these characteristics who have been identified with these predictive factors is follow-up. These patients need more attentive care than usual in the consulting room. It seems that the Spanish healthcare system does not follow global health recommendations^29^ about the prevention of suicide attempts in older adults or the elderly (thorough assessment and follow-up of anyone affected by suicidal behaviour). It is very important for healthcare professionals and others in these patients’ environments to maintain follow-up. Many suicides occur precisely in a phase of apparent improvement, when the person has the energy and will to turn desperate thoughts into self-destructive action.^1^

The study does have limitations. The age range is very wide and the number of participants may seem small; however, the study procedure did not allow for increased sample numbers or variety, as these were the people who presented to the emergency departments and met the inclusion criteria during the study period. Therefore, this type of cross-sectional research design is in itself, a limitation. However, it offers clinical profiles of interest for the prevention of repeated suicide attempt or self-injury in specific clinical subpopulations. Another limitation is that the study variables were gathered from a digital medical record that is filled in by the staff in the emergency department. This warrants a certain caution with respect to the results, although this type of procedure may be an adequate method of suicide assessment.^30^ This aspect should be standardised across emergency health services when dealing with self-destructive behaviours.

In conclusion, the need for specific health protocols for prevention of repeated self-harm and reattempts of suicide that are adapted to the psychosocial characteristics of this age group is more than evident. In fact, assessing the existence of other pre-existing risk behaviours, including previous self-injuries or suicide attempts, should be a priority for adequate health monitoring and improving social and health alert actions in older adults who are very vulnerable to suicide. There are highly predictive and modulating risk variables that can help to gauge subsequent healthcare actions so that there is a thorough follow-up after this type of behaviour. In addition, mental health professionals (psychiatrists or psychologists) need to maintain a continuous presence in emergency services, to adequately assess all of these risk variables.

## Data Availability

Anonymised data that support the findings of this study are available from the corresponding author, M.A.R.-B., upon reasonable request.
